# The role of hydrophobicity in tuberculosis evolution and pathogenicity

**DOI:** 10.1038/s41598-017-01501-0

**Published:** 2017-05-02

**Authors:** Monika Jankute, Vijayashankar Nataraj, Oona Y. -C. Lee, Houdini H. T. Wu, Malin Ridell, Natalie J. Garton, Michael R. Barer, David E. Minnikin, Apoorva Bhatt, Gurdyal S. Besra

**Affiliations:** 1 Institute of Microbiology and Infection, School of Biosciences, University of Birmingham, Edgbaston, Birmingham UK; 2 Department of Microbiology and Immunology, Institute of Biomedicine, University of Gothenburg, Gothenburg, Sweden; 3 Department of Infection, Immunity and Inflammation, University of Leicester, Leicester, UK

## Abstract

The evolution of tubercle bacilli parallels a route from environmental *Mycobacterium kansasii*, through intermediate “*Mycobacterium canettii*”, to the modern *Mycobacterium tuberculosis* complex. Cell envelope outer membrane lipids change systematically from hydrophilic lipooligosaccharides and phenolic glycolipids to hydrophobic phthiocerol dimycocerosates, di- and pentaacyl trehaloses and sulfoglycolipids. Such lipid changes point to a hydrophobic phenotype for *M. tuberculosis sensu stricto*. Using Congo Red staining and hexadecane-aqueous buffer partitioning, the hydrophobicity of rough morphology *M. tuberculosis* and *Mycobacterium bovis* strains was greater than smooth “*M. canettii*” and *M. kansasii*. Killed mycobacteria maintained differential hydrophobicity but defatted cells were similar, indicating that outer membrane lipids govern overall hydrophobicity. A rough *M. tuberculosis* H37Rv Δ*papA1* sulfoglycolipid-deficient mutant had significantly diminished Congo Red uptake though hexadecane-aqueous buffer partitioning was similar to H37Rv. An *M. kansasii*, Δ*MKAN27435* partially lipooligosaccharide-deficient mutant absorbed marginally more Congo Red dye than the parent strain but was comparable in partition experiments. In evolving from ancestral mycobacteria, related to “*M. canettii*” and *M. kansasii*, modern *M. tuberculosis* probably became more hydrophobic by increasing the proportion of less polar lipids in the outer membrane. Importantly, such a change would enhance the capability for aerosol transmission, affecting virulence and pathogenicity.

## Introduction

Coherent explanations are required for the development and upsurge in tuberculosis (TB) during the recent Holocene epoch. Developing evidence indicates an evolutionary pathway initiating from an environmental organism, possibly related to *Mycobacterium kansasii*, leading to an intermediate taxon similar to unusual smooth morphology tubercle bacilli, labelled “*Mycobacterium canettii*”^1–4^. Tuberculosis, due to *“M. canettii”*, is principally restricted to the Horn of Africa and its transmissibility is reduced in comparison with modern *Mycobacterium tuberculosis* ^5–8^. Detailed genomic scrutiny of *“M. canettii”* isolates revealed wide genetic diversity in comparison with modern members of the *M. tuberculosis* complex that are closely related^5, 9–12^.

**Figure 1:**
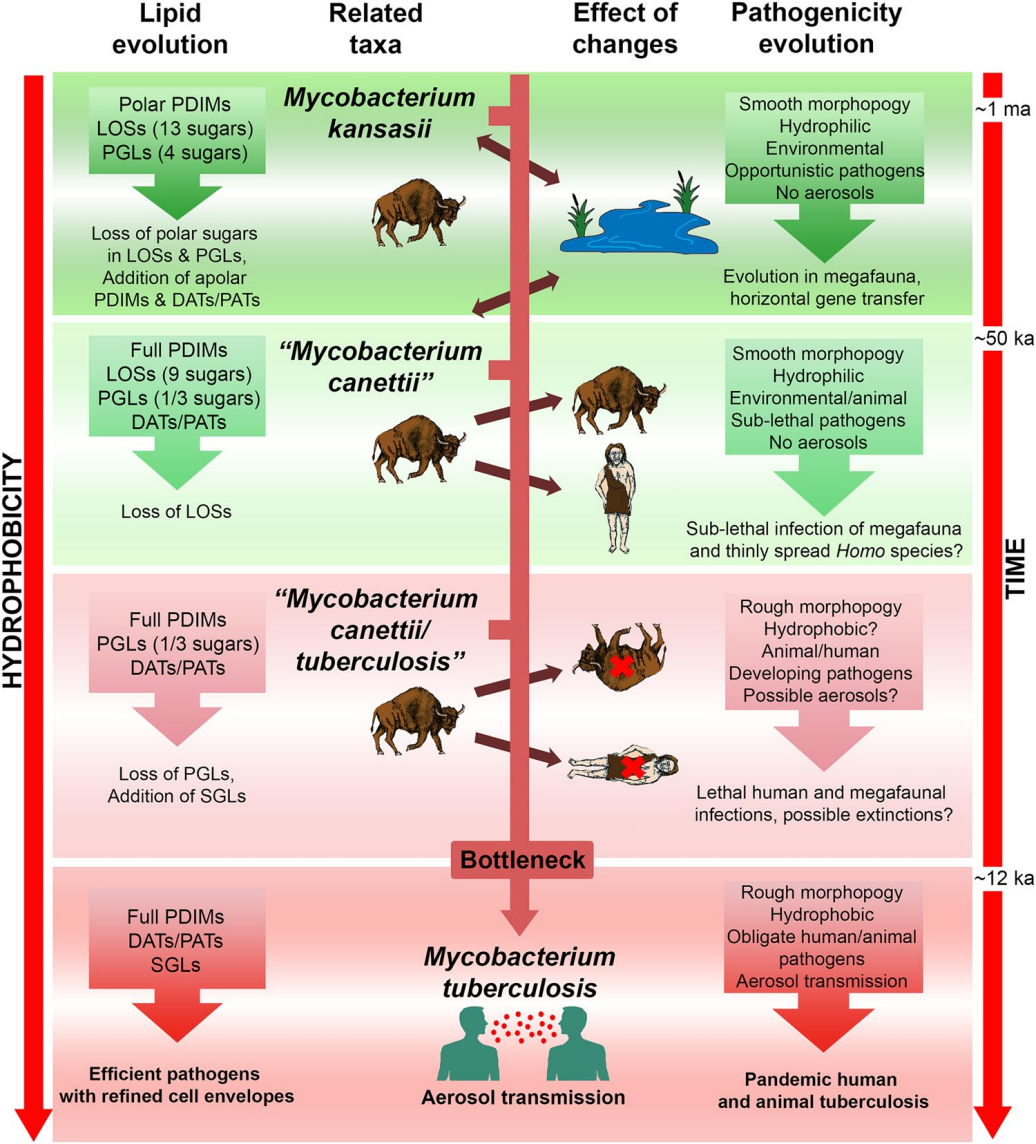
The importance of lipid composition and hydrophobicity in the evolution of tubercle bacilli. Abbreviations: PDIMs, phthiocerol dimycocerosates; LOSs, lipooligosaccharides, PGLs, glycosyl phenolphthiocerol dimycocerosates (phenolic glycolipids); DATs & PATs, di- & pentaacyl trehaloses; SGLs, sulfoglycolipids. Graded green and red backgrounds indicate environmental and mammalian pathogen associations, respectively. Evolution cannot take place directly from modern *M. kansasii* and “*M. canettii*”, which are shown as related taxa. The hypothetical transitional taxon, labelled “*Mycobacterium canettii/tuberculosis*”, possibly corresponds to the well-characterised So93R rough variant of “*M. canettii*”^29^. Increasing hydrophobicity, with time, applies to both lipid composition and whole cells.

Assuming a working hypothesis, involving an evolutionary progression from *M. kansasii*-like mycobacteria to *M. tuberculosis* via ancient relatives of *“M. canettii”*, it is possible to envisage a coherent developing pattern of mycobacterial outer membrane lipid changes (Fig. 1)^2, 3^. A strong phylogenetic link between *M. kansasii* and “*M. canettii”* is demonstrated by highly similar glycosyl phenolphthiocerol dimycocerosates, the so-called phenolic glycolipids (PGLs)^1, 13^. The main tetraglycosyl PGL of *M. kansasii* simply loses one or three sugars to produce the characteristic PGLs of “*M. canettii*”^1–3, 13^. A highly significant difference between *M. kansasii* and “*M. canettii”* resides in the so-called α-mycolic acids; subtle structural changes contribute to modified outer membrane physical properties^2, 3^. The phthiodiolone dimycocerosate waxes in *M. kansasii* are also replaced in *“M. canettii”* by a full set of dimycocerosates of the phthiocerol family (PDIMs). Distinct polar hydrophilic lipooligosaccharides (LOSs) are found in both *M. kansasii* and *“M. canettii”*, but only the latter express di- and pentaacyl trehaloses (DATs and PATs)^6^. On proceeding from *“M. canettii”* to *M. tuberculosis sensu stricto*, DATs, PATs, PDIMs are maintained, sulfoglycolipids (SGLs) are expressed, but PGLs and LOSs are deleted. The outcome of these changes is that *M. tuberculosis sensu stricto* has rather simplified mycobacterial outer membrane (MOM) free lipids, comprising only PDIMs, SGLs and DATs and PATs (Fig. 1).

It was realised that this selection of *M. tuberculosis* MOM free lipids (PDIMs, SGLs and DATs and PATs) was markedly apolar in comparison with those found in *M. kansasii* and *“M. canettii”* and this might result in relatively increased hydrophobicity of the cell surface^3^. Importantly, in other contexts, it has been demonstrated that aerosol transmissibility of non-tuberculous mycobacteria is enhanced by higher hydrophobicity^14^. Preliminary experiments^3, 15^, assessing Congo Red dye uptake^16^ and hexadecane/aqueous buffer partitioning^17^, did indeed show highly enhanced hydrophobicity of *M. tuberculosis* H37Rv.

The present study reinforces the evidence that mycobacterial strains with rough colony morphology, such as *M*. *tuberculosis* and *M*. *bovis* Ravenel, are significantly more hydrophobic than the representatives of smooth colony *M*. *kansasii* and “*M. canettii*”. Importantly, on removing the mycobacterial outer membrane lipids, the relative hydrophobicity of rough and smooth mycobacterial variants became marginal. In addition, available *M*. *tuberculosis* and *M*. *kansasii* mutants lacking SGLs and higher subclasses of LOSs, respectively^18, 19^, were used to investigate possible involvement of these lipids in cell-surface variation and hydrophobicity.

## Results and Discussion

### Lipid composition of test strains

The extractable free lipid compositions of the cultures examined are summarised in Table 1 and two-dimensional thin-layer chromatographic (2D TLC) patterns are displayed in Supplementary Figs S1–S3. Key confirmatory findings are the presence of the PDIM family in *M. tuberculosis, M. bovis* and *“M. canettii”*, with *M. kansasii* restricted to phthiodiolone dimycocerosates (Table 1, Supplementary Fig. S1). Small proportions of pentaacyl trehaloses (PATs) were detected in all except *M. kansasii* (Table 1, Supplementary Fig. S1). The expected phenolic glycolipids (PGLs) were only in *M. bovis*, “*M. canettii*” and *M. kansasii* and sulfoglycolipids (SGLs) were restricted to wild-type *M. tuberculosis* H37Rv and CDC1551, but obviously lacking in the Δ*papA1* mutant of H37Rv (Table 1, Supplementary Fig. S2). Diacyl trehaloses (DATs) were in all *M. tuberculosis* and “*M. canettii*” strains and lipooligosaccharides (LOSs) characterised *“M. canettii”* and *M. kansasii*, with a truncated range being detected in the Δ*MKAN27435* mutant of the latter (Table 1, Supplementary Fig. S3). Consistent patterns of mycolic acid methyl esters were shown for defatted cells, remaining after the extraction of free lipids (Supplementary Fig. S4). The expected α-, methoxy- and ketomycolates varied in their proportions in line with a previous study^20^. Notably, gene knockouts did not change the general mycolic acid patterns found for *M. tuberculosis* H37Rv and *M. kansasii* (Supplementary Fig. S4).

**Table 1:** Distribution of cell envelope lipids in test strains.

	PDIM	TAG	PAT	DAT	PGL(1)	PGL(>1)	SGL	LOS
*M. tuberculosis* H37Rv	+++	++	+	+	−	−	+	−
*M. tuberculosis* H37Rv Δ*papA1*	+++	++	+	+	−	−	−	−
*M. tb* CDC1551	+++	++	+	+	−	−	+	−
*M. bovis* Ravenel	+++	++	+	−	+*	−	−	−
*“M. canettii”* 140010060	+++	+	+	+	+*	+***	−	++
*“M. canettii”* 140010061	+++	+	+	+	+*	+***	−	++
*M. kansasii* Hauduroy	++^#^	++	−	−	−	+****	−	++
*M. kansasii* Δ*MKAN27435*	++^#^	++	−	−	−	+****	−	+

Abbreviations: PDIM, dimycocerosates of phthiocerol A, phthiocerol B and phthiodiolone (++^#^Indicates phthiodiolone); TAG, triacylglycerols; PAT & DAT, penta- and diacyl trehaloses; PGL(1), single sugar phenolic glycolipid (+*); PGL(>1), multiple sugar phenolic glycolipid (+*** & +****, 3 & 4 sugars); SGL, sulfoglycolipid; LOS, lipooligosaccharides.

### Relative hydrophobicity of test strains

Two complementary methods were employed to determine the relative hydrophobicity of *M. kansasii* and members of the *M. tuberculosis* complex, including “*M. canettii*”. The Congo Red procedure involves an amphiphilic dye that associates with lipophilic regions of the mycobacterial cell envelope, providing a measure of overall hydrophobicity^16, 21–23^. An alternative approach partitions cells between a hexadecane organic phase and an aqueous buffer^17, 21–24^. In this study, Tween 80 was included in liquid culture to ensure uniform comparable growth and cell envelope lipid composition; it is likely that any loosely attached capsular material may have been removed^25^. Preliminary studies, using these procedures, have demonstrated that the rough colony morphology H37Rv type strain of *M. tuberculosis* is much more hydrophobic than the smooth colony morphology representatives of “*M. canettii*” and *M. kansasii* ^3, 15^.

**Figure 2:**
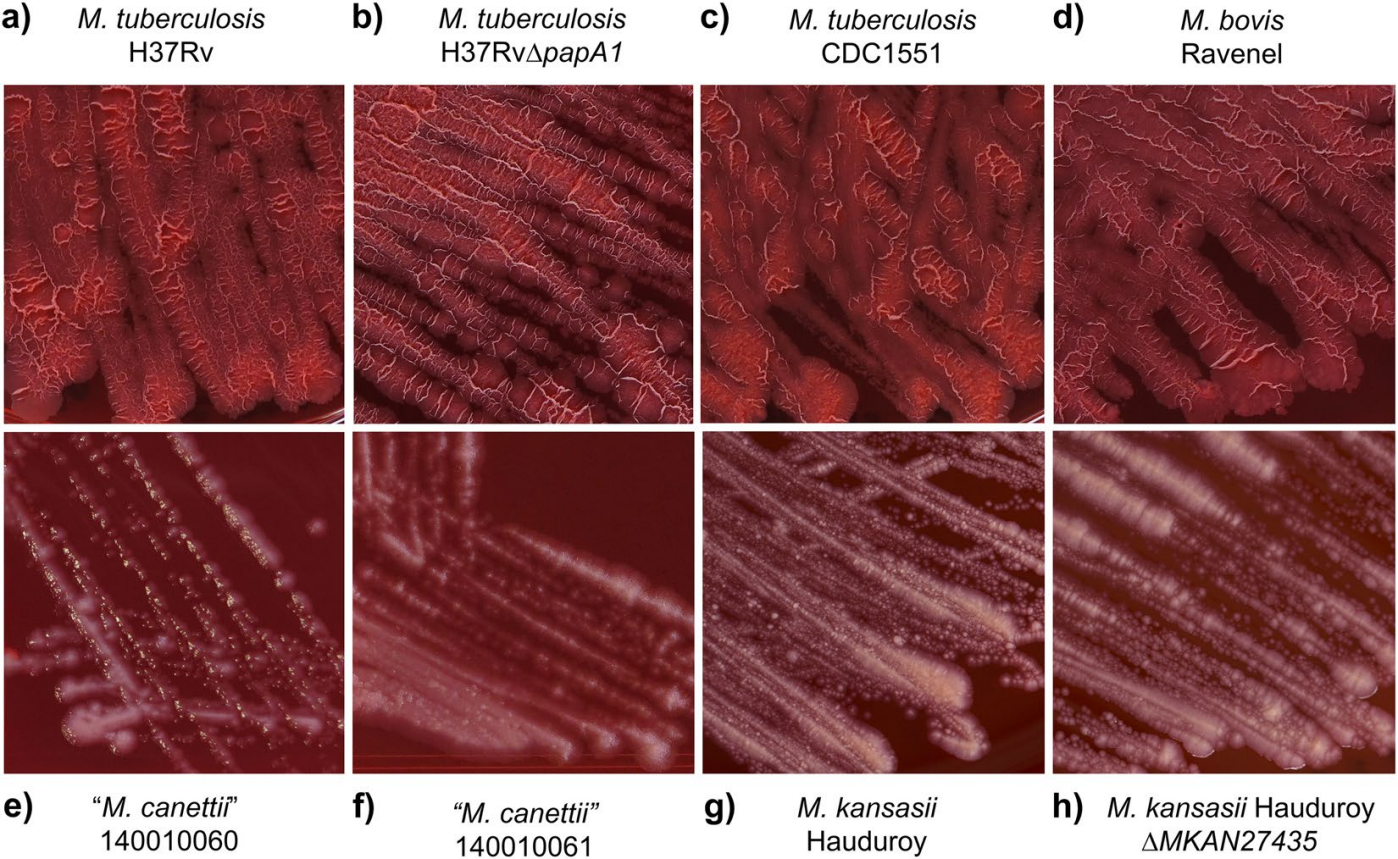
Staining of test strains with Congo Red dye on solid media. (**a**) *M. tuberculosis* H37Rv; (**b**) *M. tuberculosis* H37Rv Δ*papA1*; (**c**) *M. tuberculosis* CDC1551; (**d**) *M. bovis* Ravenel; (**e**)“*M. canettii”* 140010060; (**f**) “*M. canettii*” 140010061; (**g**) *M. kansasii* Hauduroy; (**h**) *M. kansasii* Hauduroy Δ*MKAN27435*.

**Figure 3:**
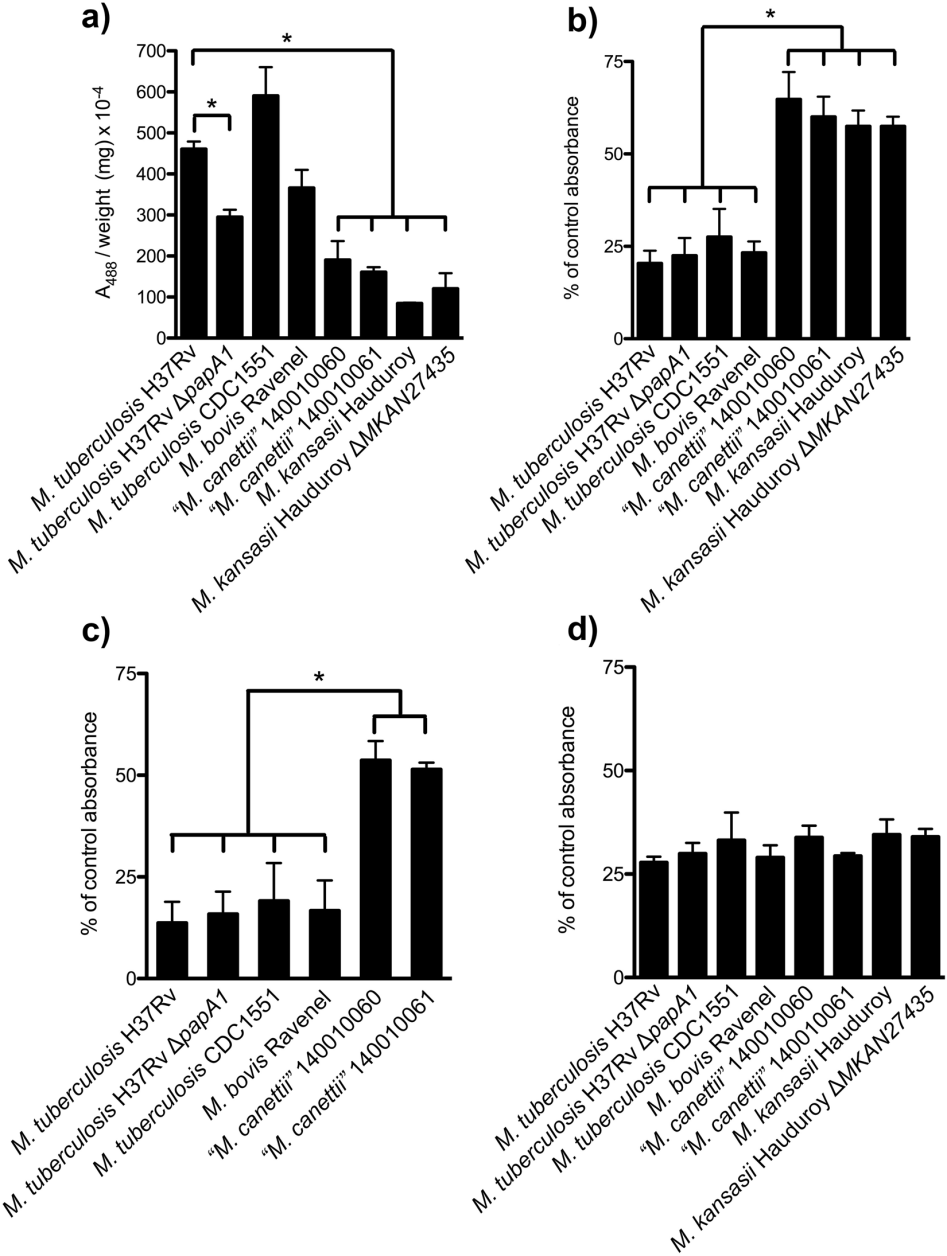
Quantitative assessment of relative mycobacterial hydrophobicity. (**a**) Quantification of Congo Red extracted from solid media growth. Hexadecane/aqueous buffer partitioning of: **(b)** Live cells, (**c**) Killed cells, (**d**) Defatted killed cells. Results are expressed as mean ± standard deviation of at least three independent experiments. Statistical significance was determined by Student’s *t*-test (p < 0.01).

Cultivation in the presence of Congo Red (Fig. 2) showed rough colonies for *M. tuberculosis* H37Rv, CDC1551 and *M. bovis* Ravenel, with clear dye uptake (Fig. 2a–d). The rough morphology of *M. bovis* Ravenel is characteristic of laboratory-adapted strains. The SGL-deficient Δ*papA1* mutant of *M. tuberculosis* H37Rv remained rough and absorbed dye uniformly (Fig. 2b). In contrast, smooth colonies of *“M. canettii”* and *M. kansasii* were poorly stained with Congo Red, as was the Δ*MKAN27435* mutant that was partially deficient in LOS production (Table 1, Supplementary Fig. S3) but remained smooth (Fig. 2e–h). The quantitative results for the amount of Congo Red dye extracted are summarised in Fig. 3a and full numerical data are provided in Supplementary Table S1. The two *M. tuberculosis* strains absorbed the most dye, with CDC 1551 being the strongest. The H37Rv Δ*papA1*mutant significantly took up less dye than the parent strain and *M. bovis* Ravenel had lower dye uptake than *M. tuberculosis*. As indicated previously^3, 15^, the two strains of “*M. canettii*” absorbed much less dye than the *M. tuberculosis* representatives. The dye absorbed by *M. kansasii* was reduced in comparison with the “*M. canettii”* strains, but the Δ*MKAN27435* partially LOS-deficient mutant appeared to absorb more dye than the parent. The statistically significant reduced Congo Red uptake of the H37Rv Δ*papA1* mutant (Fig. 3a) could be correlated with loss of sulfoglycolipid (SGL) (Table 1, Supplementary Fig. S2), with its extremely hydrophobic phthioceranate and hydroxyphthioceranic fatty acids^3^. Conversely, diminished hydrophilic LOS content (Table 1, Supplementary Fig. S3) did not give a decisive increase in dye retention in *M. kansasii* Δ*MKAN27435* (Fig. 3a).

Partitioning of live cultures between hexadecane and aqueous buffer again revealed the presence of two clear categories (Fig. 3b). *M. bovis* Ravenel, the two *M. tuberculosis* strains and the Δ*papA1* mutant of H37Rv were significantly more hydrophobic in comparison with the “*M. canettii*” and *M. kansasii* strains, with the latter taxa having practically doubled affinities for the aqueous phase. Interestingly, *M. tuberculosis* CDC1551 was marginally less hydrophobic than *M. tuberculosis* H37Rv, the H37Rv Δ*papA1* mutant and *M. bovis*. The *M. kansasii* Δ*MKAN27435* mutant was not distinguishable from its parent strain (Fig. 3b). The partitioning of representative killed cultures gave discriminatory results (Fig. 3c), paralleling those for live cells (Fig. 3b), but with reduced overall hydrophilicity. Partition analysis of delipidated cells (Fig. 3d) gave relatively uniform results, with comparable hydrophobicities. Numerical data for partitioning studies are included in Supplementary Table S2.

The Congo Red and hexadecane-aqueous buffer partitioning techniques showed encouraging correlations, but certain subtle differences were apparent. In the partitioning studies, *M*. *tuberculosis* CDC1551 was marginally more hydrophilic than *M*. *tuberculosis* H37Rv (Fig. 3b), in contrast to the results of the Congo Red dye uptake (Fig. 3a). A previous study also found that *M. tuberculosis* CDC1551 was more hydrophilic in partition experiments than *M. tuberculosis* H37Rv, but hydrophobic parity was achieved by introducing a second copy of the *phoPR* operon into CDC1551^26^. Lipid profiles were not recorded in that study, but PhoPR is known to regulate cell envelope phenotypes, including synthesis of DATs, PATs and SGLs^27^. The Δ*papA1* mutant was only marginally more hydrophilic than the parent *M. tuberculosis* H37Rv in partition experiments (Fig. 3b), when it was significantly greater with Congo Red (Fig. 3a). *M*. *bovis* Ravenel was intermediate in partition experiments between the two representatives of *M. tuberculosis*. The diminished LOS content of the *M. kansasii* Δ*MKAN27435* mutant, that remained smooth, had no significant effect on partitioning between the aqueous and organic phases; however, it should be noted that the polar PGL content was maintained in both cultures (Table 1, Supplementary Fig. S2). It appears that total loss of polar LOSs from smooth *M. kansasii* may be necessary to generate a rough variant, as shown previously^28^. A spontaneous mutant of smooth “*M*. *canettii*”, totally deficient in LOSs, was transformed to rough morphology even though it maintained the presence of polar PGLs^29^.

The differential hydrophobic properties of the rough and smooth strains, under study, are most clearly demonstrated by partitioning between hexadecane and aqueous buffer (Fig. 3b) rather than by Congo Red uptake on solid media (Fig. 3a). The partitioning experiment probably reflects the gross physical properties of the relatively homogeneous liquid-grown biomass. In contrast, the Congo Red staining involves more heterogeneous solid surface cultures that may include differentially responsive variations between individual cells. Overall uptake of Congo Red dye will represent the sum of many individual micro interactions in cells having subtle compositional differences.

Partitioning a selection of killed biomass (Fig. 3c) demonstrated that the effects, chronicled so far, reflect innate properties of the cells, independent of viability. The dead cells were somewhat more hydrophobic than the live equivalents but the relative hydrophobicities remained the same (Fig. 3b,c). Perhaps immobilisation of the envelope lipids resulted in some consolidation of the cell surface, this being particularly apparent for the Δ*papA1* mutant of *M. tuberculosis* H37Rv (Fig. 3c). Stripping away the extractable cell envelope lipids produced dead cell cores, all with comparable hydrophobicities (Fig. 3d). This is a good indication that variations in cell surface hydrophobicity are governed by free lipid composition, particularly those residing in the mycobacterial outer membrane. In the case of the H37Rv Δ*papA1* mutant, the dead cell core was of comparable hydrophobicity (Fig. 3d), suggesting that any perturbations affecting physical properties were limited to the outer membrane. It is notable that the ~25% hydrophilicity of the delipidated cells (Fig. 3d) is greater than that (<10%) recorded for killed *M. tuberculosis* and *M. bovis* Ravenel (Fig. 3c), signifying that key non-polar hydrophobic lipids have been sacrificed. Conversely, removal of relatively polar and hydrophilic lipids, such as LOSs and PGLs, will reduce the hydrophilicity of killed *“M. canettii”* from approximately 50% (Fig. 3c) to the common ~25% level of the delipidated cells (Fig. 3d). The overall uniform level of the relatively high hydrophobicity of the delipidated cells presumably is an expression of the presence of covalently-bound 70–90 carbon mycolic acids. Such mycolic acids assemble in a monolayer linked to arabinogalactan, with the distal portions interacting with a range of characteristic free lipids to create a coherent mycobacterial outer membrane^3, 30^. Released from the restraints of free lipid interactions, presumably all the mycolate distal hydrocarbon chains collapse to provide a relatively uniform hydrophobic external layer.

As previously noted^3^, the enhanced hydrophobicity of the representatives of *M. tuberculosis sensu stricto* appears to reside in the cell envelope free lipids (Fig. 1). The principal outer membrane free lipids of *M. kansasii* are a limited selection of relatively polar phenolic glycolipids (PGLs) and lipooligosaccharides (LOSs)^2, 3^, accompanied by a restricted phthiodiolone version of the phthiocerol dimycocerosate (PDIM) family (Table 1, Supplementary Figs S1–S3). The intermediate *“M. canettii”* taxon has an extended cocktail of outer mebrane free lipids, adding DATs, PATs and a full complement of the PDIM family, in addition to PGLs and LOSs. The loss of the ability to synthesise polar hydrophilic PGLs and LOSs, coupled with SGL production, gave the refined MOM free lipid composition characteristic of *M. tuberculosis* (Fig. 1)^2, 3^. Even though the trehalose-based SGLs include a polar sulfate functionality, the distinctive very long-chain phthioceranate and hydroxyphthioceranate fatty acid constituents possibly contribute to increased hydrophobicity of the outer membrane surface. From the point of view of the cell envelope lipophilic components, it is clear that *M. kansasii* and *M. tuberculosis* are adapted to their particular stable environments, but *“M. canettii”* may represent a transitory state suited to variable evolutionary situations^2, 3^. A major area of uncertainty is the role of triacylglycerols (TAGs) in the mycobacterial outer membrane. It was shown by selective extraction that substantial amounts of TAGs are apparently located in the outer membrane of *Mycobacterium smegmatis* ^31^, but the precise molecular structures remain to be delineated. Certain mycobacteria display substantial TAG-rich lipid bodies that are implicated in energy storage^32^.

### Lipid composition and rough and smooth morphology of mycobacteria

A smooth to rough change in a scotochromogenic *Mycobacterium* correlated with loss of a glycopeptidolipid^33^ and this was confirmed in *Mycobacterium intracellulare* ^34^. Rough variants of smooth *M. kansasii* lacked polar lipooligosaccharides^28^ and correlation with a previous study^35^ indicated that smooth strains were more rapidly cleared from mice organs. A rough variant (So93R) of *“M. canettii”* did not revert to its smooth progenitor So93; it had lost the capacity to produce lipooligosaccharides (LOSs) and it was significantly more virulent in the Guinea pig^29^. The rough So93R variant retained the triglycosyl phenolic glycolipid PGL^29^. Knocking out the gene for the production of LOSs converts smooth *“M. canettii”* strains to rough variants^12^. A rough mutant of *M. smegmatis* lacking glycopeptidolipids, had increased cell hydrophobicity, clumped to form aggregates and was rapidly phagocytosised by human macrophages^21^. Surface glycopeptidolipids were found to inhibit human macrophage phagocytosis of *M. smegmatis* ^36^. A rough *M. marinum* mutant, lacking LOSs, was phagocytosed more efficiently^37^.

### Hydrophobicity and the pathogenicity of *Mycobacterium abscessus*

*Mycobacterium abscessus* is emerging as an important cause of pulmonary infections in cystic fibrosis patients^38^. It was found that smooth strains were cleared, but rough variants persisted and multiplied in a murine pulmonary infection model^39^. The smooth (390S) and rough (390R) colonies were moist and dry, respectively, correlating with hydrophilicity and hydrophobicity. Only the rough variants exhibited cording, drawing comparison with virulent *M. tuberculosis* ^39^. A follow-up study confirmed cording and proved that rough *M. abscessus* lacked glycopeptidolipids (GPLs)^40^. The same phenotypes were found for rough and smooth *M. abscessus*, including two of the same strains, 390R and 390S; another rough variant was hyper-lethal for mice^41^. The emergence of an isogenic rough variant of *M. abscessus* resulted in acute respiratory failure^42^. Rough variants of *M. abscessus* infected or colonized the airways of cystic fibrosis patients and some isolates were harboured for years^43^. In contrast, contaminants and wound isolates mainly exhibited smooth colony morphology^43^. Smooth *M. abscessus* was internalised by human monocytes but rough variants were not, as they formed serpentine cords^44^. The lack of GPLs could make rough strains cord together and be difficult to internalise^44^. Using the zebrafish model, rough *M. abscessus* formed cords that prevented phagocytosis, initiated abscess formation and rapid larval death^45^. A rough variant, deficient in GPL production/transport, produced cords *in vitro* ^46^. A comprehensive investigation of smooth-to-rough morphotype alterations implicated lipid changes in cellular interactions of clinically persistent *M. abscessus* ^47^. Rough morphology *M. abscessus* strains, from lung infections, correlated with shifts in lipid metabolism indicating triacylglycerol accumulation and some GPL loss^48^. Greater host inflammatory responses were seen for rough *M. abscessus* morphotypes in a murine model^49^. Expanding on previous cording studies^50, 51^, it was found that clumped rough *M. abscessus* variants had increased phagocytosis and damaged macrophages^52^.

### Hydrophobicity and aerosol transmission

As pointed out previously^3, 15^, a very significant implication of enhanced mycobacterial hydrophobicity is that the propensity for aerosol transmission is facilitated. This principle has been established in extended investigations of members of the *Mycobacterium avium* family and other nontuberculous mycobacteria, where clear correlations were demonstrated between hydrophobicity and entry into aerosols from aqueous media^14, 53, 54^. The significance of the clearly enhanced hydrophobicity of *M. tuberculosis* is the implication that aerosol transmission would be encouraged^3, 15^. Similarly, it was noted^3^ that rough variants of *M. abscessus*, lacking polar glycopeptidolipids are likely to be more hydrophobic and aerosol transmissible than the smooth strains. As summarised above, the overall virulence of rough *M. abscessus* strains is apparently enhanced and it was also noted that isolates causing chronic airway colonisation/infection are usually rough^43, 44, 48^. In a comprehensive world-wide survey of *M. abscessus* infections, the possibility of aerosol transmission was raised but any link with rough-smooth variations was not addressed^38^. The degree of hydrophobicity and propensity for aerosol transmission of rough *M. abscessus* variants requires detailed investigation.

### Evolutionary origins of pathogenic rough tubercle bacilli

It is important to consider when less virulent smooth tubercle bacilli evolved into rough pathogenic members of the current *M. tuberculosis* complex. Recent genomic extrapolations claimed that TB is not older than ~6 ka^55, 56^, but this simply points to the most recent common ancestor of the specimens investigated^57, 58^. Coalescent analyses of TB and human mitochondrial genome evolution predicted that TB co-emerged with *Homo sapiens* from Africa 70,000 years ago^59^, but linked co-evolution is not a necessary consequence of parallel evolution. A consensus concludes that tubercle bacilli have passed through a bottleneck, around 35–20 ka BP^9–11, 60^. Post-bottleneck, members of the *M. tuberculosis* complex arose through linear gene deletions^61–66^. Prior to the bottleneck, there is evidence for horizontal gene transfer in ancestral strains^1, 67, 68^.

TB has been diagnosed in a ~9 ka woman and child from Atlit-Yam in the Eastern Mediterranean^69, 70^ and a ~17 ka bison from Natural Trap Cave, Wyoming^70, 71^. Clear *M. tuberculosis* complex DNA sequences were amplified, supported by lipid biomarkers^70^. The oldest TB in *H. sapiens* was recorded in Syrian skeletons from 10.8–10.3 ka, DNA amplification being backed up by lipid biomarker profiles^72^. There is a lack of evidence for tuberculosis in *H. sapiens*, prior to the Holocene, probably due to low population densities^73^. However, diagnostic bone lesions indicate tuberculosis in Pleistocene megafauna, back to ~120 ka BP^74, 75^. Diverse microbial species in megafaunal oral cavities and digestive organs would be available to participate in evolutionary horizontal gene transfer^67, 68^. A feasible scenario (Fig. 1), for the emergence of all the modern biotypes of the *M. tuberculosis* complex through the bottleneck would involve a complex web of interactions between *H. sapiens* and Pleistocene animal reservoirs until the dramatically ameliorated climatic conditions at the beginning of the Holocene allowed humans to form settlements and promulgate communal tuberculosis.

### Implications for mammalian extinctions in the Pleistocene

The accumulating evidence of TB lesions in a wide range of extinct Pleistocene megafauna^74, 75^ arouses suspicion that TB may have contributed to the extinction of particular classes of these animals (Fig. 1). Mammoths are exceptional, as they do not appear to have bone lesions, attributable to TB^74, 75^. As noted above, many of these megafauna may have survived to maturity with sub-lethal disease; an informative example is the 17 ka Natural Trap Cave mature bison^71, 76^ that was not killed by TB, as it suffered an obvious lethal accident. The possible involvement of bacterial disease in megafaunal extinctions has received less and less credence over the years, the only possibility deemed worthy of mention being unspecified “hyperdisease” contracted from man or other mammalian species^77, 78^. The hypothesis advanced here is a diametrically opposite scenario, with environmental bacteria slowly evolving into tubercle bacilli using megafaunal hosts^2, 3, 15^. Once hydrophobic tubercle bacilli became transmissible in aerosols, snorting bison and other megafauna would rapidly spread lethal disease within their own herds and greatly facilitate infection of other species. The fact that a number of megafaunal extinctions happened over a relatively short time favours the primary rapid intervention of a particular factor, such as virulent TB, rather than the more secondary gradual effects of environmental change and/or human interaction^77, 78^. Early cases of TB in *H. sapiens* in the so-called Fertile Crescent region^69, 72^ may correlate with naïve humans migrating out of Africa, at the conclusion of the Ice Ages, encountering contagious rough hydrophobic tubercle bacilli of megafaunal origin.

### Conclusions

Enhanced hydrophobicity is an important characteristic of pathogenic members of the modern *M. tuberculosis* complex. The evolutionary transformation from hydrophilic environmental low-pathogenicity ancestors, related to *M. kansasii* and *“M. canettii”*, to hydrophobic virulent tubercle bacilli is particularly decisive (Fig. 1), but it is important to be aware of zoological and paleogeographic aspects. The oldest proven cases of TB in *H. sapiens* are so far restricted to ~11–9 ka in the early Holocene, but there is developing evidence for megafaunal TB in the late Pleistocene. It is plausible for environmental mycobacteria to have experienced the necessary horizontal gene transfer in evolving into intermediate tubercle bacilli, related to *“M. canettii”*, in the multiple intestinal tracts of Northern Hemisphere Pleistocene megafauna rather than in thinly-spread human hosts. The high diversity of the *“M. canettii”* taxon might indicate that its evolution was multi-centred over an extended period resulting in animals carrying “smooth” bacteria with low pathogenicity and transmissibility. The emergence of rough *“M. canettii”* hydrophobic variants, readily transmitted in aerosols, may well have contributed to the demise of Pleistocene megafauna and any associated mammals (Fig. 1). Modern humans, settling in the Eastern Mediterranean so-called Fertile Crescent, may have encountered bottleneck-refined virulent tubercle bacilli and initiated the modern human and animal TB pandemic.

The broad conclusions concerning the influence of hydrophobicity on the evolution of tubercle bacilli are summarised in Fig. 1. The scheme is essentially self-explanatory, as it mainly illustrates the major points discussed previously. The taxon, provisionally labelled *“Mycobacterium canettii/tuberculosis”*, represents a pivotal stage in TB evolution. The relatively low-pathogenicity smooth *“M. canettii”* strains do have many attributes of modern *M. tuberculosis*, but they may have co-existed with mammalian hosts without decimating populations. Prior to the bottleneck refinement, there may have been a variety of rough tubercle bacilli with developing potential for pathogenicity and rapid aerosol transmission. The hypothetical *“Mycobacterium canettii/tuberculosis”* taxon is simply a representative of bacteria in process of developing enhanced pathogenic traits; it possibly corresponds to the well-characterised So93R rough variant of “*M. canettii*”^29^.

The relentless pathogenic success of current mycobacterial pathogens is probably a direct consequence of the special hydrophobic properties of rough variants, more virulent than corresponding smooth strains. Aerosolisation of hydrophobic bacilli is favoured and such rough organisms are more difficult to clear in animals than smooth varieties. In addition to members of the *M. tuberculosis* complex and other slow-growing mycobacterial pathogens, these principles are very applicable to the important rapid-growing strains of *M. abscessus* that are a major problem for cystic fibrosis patients.

## Methods

### Bacterial strains and culture media

Mycobacterial strains included in this study were as follows: *Mycobacterium tuberculosis* H37Rv, the SGL-deficient mutant *M*. *tuberculosis* H37Rv Δ*papA1* ^18^, *Mycobacterium tuberculosis* CDC1551, *Mycobacterium bovis* Ravenel, “*Mycobacterium canettii*” CIPT 140010060 and 140010061 (Institute Pasteur, Paris)^79^, *Mycobacterium kansasii* Hauduroy (ATCC12478), and the partial LOS-deficient *M*. *kansasii* Δ*MKAN27435* ^19^. Homogenous static liquid cultures of mycobacterial strains were prepared aerobically in shallow flat flasks with filter caps to allow gas exchange. Growth at 37 °C in 7H9 liquid media, containing 10% v/v OADC, 0.2% v/v glycerol and 0.05% v/v Tween-80, was allowed to an optical density (OD) 600 nm of 0.8–1.0 followed by harvesting at 3000 g and washing with PUM buffer (22.2 g K_2_HPO_4_ 3H_2_O, 7.26 g KH_2_PO_4_, 1.8 g urea, 0.2 g of MgSO_4_ 7H_2_O and distilled water to 1 L; pH 7.1). Cell pellet material was divided and one half was killed by autoclaving at 121 °C for 15 min. Solid media growth was on 7H10 agar plates containing 10% v/v OADC, 0.5% v/v glycerol and Congo Red at 100 µg/ml at 37 °C until bacterial colonies became clearly visible.

### Lipid extraction and analysis

Apolar and polar fractions of free lipids were recovered from 150 mg wet autoclaved biomass, washed with PBS buffer, according to an established protocol^80^. Profiles of the lipid classes were recorded by a series of two-dimensional thin-layer chromatographic (TLC) systems (Supplementary Figs S1–S3), covering the whole range of lipid polarities^80^. Apolar fraction lipids were visualised by spraying with 5% w/v ethanolic molybdophosphoric acid and charring with a heat gun. Glycolipids in polar fractions were revealed by spraying with ethanolic α-naphthol-sulfuric acid^80^, followed by gentle charring with a heat gun; the same procedure was used to confirm the presence of glycolipids in apolar fractions. Mycolic acid methyl esters were released from lyophilised defatted cells, by alkaline methanolysis^81^. Cellular material (100 mg) was placed in a 10 ml PTFE capped glass tube and 0.5 % methanolic potassium hydroxide (2 ml) was added. The mixture was kept at 37 °C for 3 days. Cell residues were washed twice with methanol (2 ml) to remove all lipids, excepting methanol-insoluble mycolates. Mycolic acid methyl esters (MAMEs) were extracted from the residues with diethyl ether (3 × 1 ml). Samples were subjected to TLC, using silica gel plates (5725 silica gel 60F254, Merck), developing thrice in petroleum ether (60–80 °C)/diethyl ether (90:10). MAMEs were visualised by spraying with 5% w/v ethanolic molybdophosphoric acid, followed by charring with a heat gun (Supplementary Fig. S4). TLC plates were scanned using CanoScan LiDE 220 scanner and images were processed using Adobe Photoshop CC 2015 software.

### Congo Red binding assay

Cells scraped from Congo Red plates were washed with distilled water until the supernatant was colourless and re-suspended in 3 ml of dimethyl sulfoxide (DMSO). After mixing for 3 h at room temperature, cells were harvested by centrifugation (20 min at 3000 g) and the extraction step repeated twice. Pooled supernatants were dried under vacuum and re-dissolved in 1 ml of DMSO; Congo Red was spectrophotometrically measured at OD 488 nm. Congo Red binding index was defined as A_488_ of the DMSO extracts divided by the dry weight (mg) of the cell pellet. Results are expressed as mean ±SD of at least three independent experiments. Photographs of agar plates were taken with a Canon PowerShot G16 and Canon EOS 5D Mark III.

### Hexadecane-aqueous buffer partitioning

Live exponentially growing cells from liquid culture were washed twice in PUM buffer and finally suspended to an OD of 0.7 at 600 nm. Aliquots (3 ml) were transferred to glass tubes and hexadecane (2.4 ml) added. After brief mixing, samples were incubated for 8 min at 37 °C and phase separation allowed at 22 °C for 15 min. Hydrophobicity index was defined as aqueous phase OD at 600 nm, expressed as a percentage of that of the bacterial suspension in PUM buffer alone. Results are expressed as mean ±SD of at least three independent experiments. The same procedure was applied to a selection of autoclaved cells and cell core material after extraction of lipids, as above.

### Statistical analysis

The results were expressed as mean ± SD from at least three independent experiments. The student’s unpaired t-test (equal variance assumed) was used to evaluate differences between the samples. Two-tailed P-values below 0.01 were considered statistically significant. Graphic data were prepared with GraphPad Prism software.

## Electronic supplementary material

Supplementary Information
