# Supplementary material for: The role of hydrophobicity in tuberculosis evolution and pathogenicity
Source: Sci Rep. 2017 May 2;7:1315. doi: 10.1038/s41598-017-01501-0 (PMC5431016; doi:10.1038/s41598-017-01501-0)
Supplement: Supplementary file 1 — Supplementary Information [file 41598_2017_1501_MOESM1_ESM.pdf]

SUPPLEMENTARY INFORMATION

The role of hydrophobicity in tuberculosis evolution and pathogenicity

Monika Jankute,[1] Vijayashankar Nataraj,[1] Oona Y-C. Lee,[1] Houdini H.T. Wu,[1] Malin Ridell,[2] Natalie J. Garton,[3] Michael R. Barer,[3] David E. Minnikin,[1] Apoorva Bhatt[1] and Gurdyal S. Besra[1,*]

[1]Institute of Microbiology and Infection, School of Biosciences, University of Birmingham, Edgbaston, Birmingham, UK.

[2]Department of Microbiology and Immunology, Institute of Biomedicine, University of Gothenburg, Gothenburg, Sweden.

[3]Department of Infection, Immunity and Inflammation, University of Leicester, Leicester, UK.

Table of contents:

Supplementary Tables p2

Supplementary Figures p4

References p9

SUPPLEMENTARY TABLES

Supplementary Table S1. Congo Red binding by various *Mycobacterium* strains

Species	Strain	Colony phenotype	Results<br>Mean (A488/weight mg x 10^{-4}) ± SD*
M. tuberculosis	H37Rv	Rough	460.6 ± 18.3
M. tuberculosis	H37Rv Δ*papA1*	Rough	294.6 ± 18.2
M. tuberculosis	CDC1551	Rough	590.2 ± 70.4
M. bovis	Ravenel	Rough	365.8 ± 44.4
“M. canettii”	140010060	Smooth	190.1 ± 46.2
“M. canettii”	140010061	Smooth	160.8 ± 12.0
M. kansasii	Hauduroy	Smooth	84.5 ± 1.9
M. kansasii	Hauduroy Δ*MKAN27435*	Smooth	120.5 ± 37.7

*Relative Congo red binding by mycobacteria grown on agar was determined as described in materials and methods. Data represent mean ± SD from three independent experiments.

Supplementary Table S2. Hexadecane-aqueous buffer partitioning of various *Mycobacterium* strains

Species	Strains	Colony phenotype	Results: Mean (% of control absorbance) ± SD*		
			Live bacteria	Heat killed bacteria	Delipidated bacteria
M. tuberculosis	H37Rv	Rough	20.4 ± 3.4	13.7 ± 5.2	27.8 ± 1.4
M. tuberculosis	H37Rv Δ*papA1*	Rough	22.5 ± 4.8	15.9 ± 5.5	29.9 ± 2.6
M. tuberculosis	CDC1551	Rough	27.5 ± 7.6	19.0 ± 9.3	33.2 ± 6.7
M. bovis	Ravenel	Rough	23.3 ± 3.1	16.7 ± 7.5	29.0 ± 3.0
"M. canettii"	140010060	Smooth	64.8 ± 7.4	53.7 ± 4.8	33.8 ± 2.9
"M. canettii"	140010061	Smooth	60.0 ± 5.5	51.4 ± 1.6	29.4 ± 0.7
M. kansasii	Hauduroy	Smooth	57.5 ± 4.3	ND	34.5 ± 3.7
M. kansasii	Hauduroy Δ*MKAN27435*	Smooth	57.5 ± 2.6	ND	34.0 ± 1.9

*Relative hydrophobicity was tested as described in materials and methods. Data represent mean ± SD from three independent experiments.
ND: Not determined

SUPPLEMENTARY FIGURES

Supplementary Figure S1

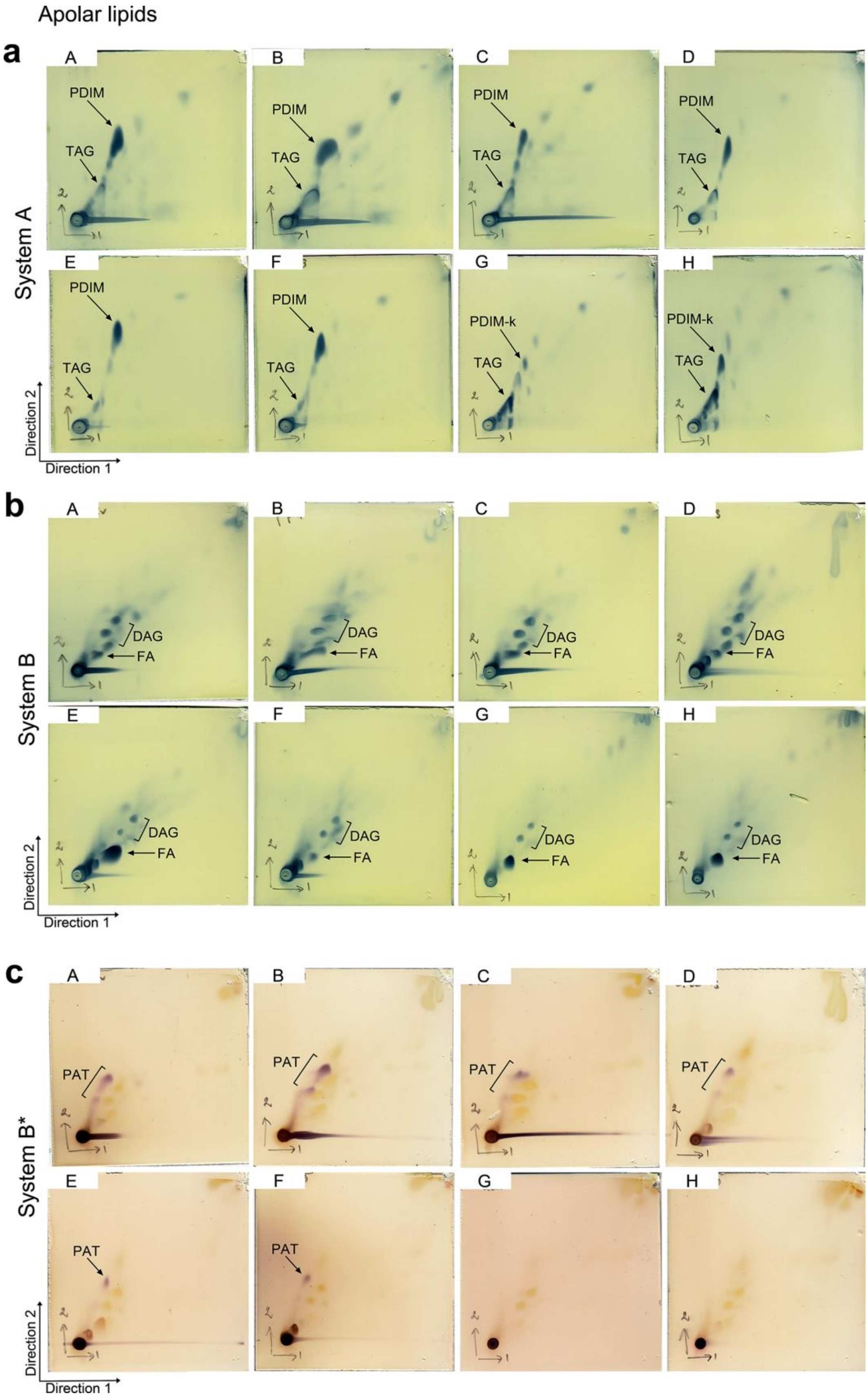

Supplementary Figure S1. Thin layer chromatography (TLC) patterns of least polar lipids from apolar lipid fractions extracted from various *Mycobacterium* strains. (A) *Mycobacterium tuberculosis* H37Rv, (B) *M. tuberculosis* H37Rv Δ*papA1*, (C) *M. tuberculosis* CDC1551, (D) *Mycobacterium bovis* Ravenel, (E) "*Mycobacterium canettii*" 140010060, (F) "*M. canettii*" 140010061, (G) *Mycobacterium kansasii* Hauduroy, (H) *M. kansasii* Hauduroy Δ*MKAN27435*. Lipid fractions were run as previously described by Dobson *et al*., 1985[1] using the following solvent systems:

(**a**) System A. Molybdophosphoric acid detection: *all lipids*.

Direction 1: Petroleum ether/ethyl acetate (98:2, v/v, thrice);

Direction 2: Petroleum ether/acetone (98:2, v/v, once)

(**b**) System B. Molybdophosphoric acid detection: *all lipids*.

Direction 1: Petroleum ether/acetone (98:2, v/v, thrice);

Direction 2: Toluene/acetone (95:5, v/v, once)

(**c**) System B. α-Naphthol-sulfuric acid detection: *glycolipids*.

Abbreviations: PDIM, dimycocerosates of phthiocerol A, phthiocerol B and phthiodiolone (characteristic of *M. tuberculosis* complex); PDIM-k, dimycocerosates of phthiodolone (characteristic of *M. kansasii*); TAG, triacylglycerols; DAG, diacylglycerols; FA, free fatty acids; PAT, pentaacyl trehaloses (obscured by other lipids in **b**, but revealed in **c** with glycolipid spray).

Supplementary Figure S2.

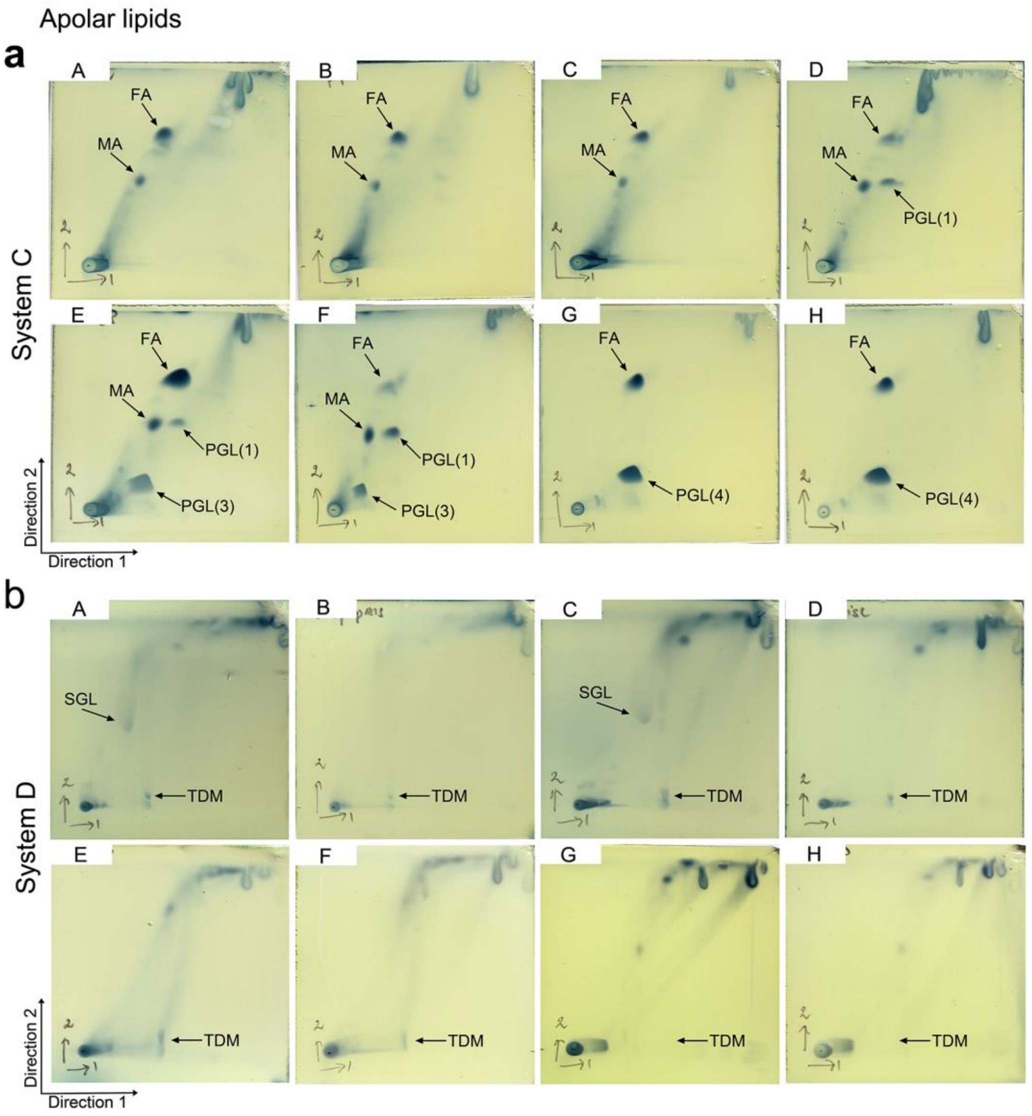

Supplementary Figure S2. Thin layer chromatography (TLC) patterns of relatively polar lipids from apolar lipid fractions extracted from various *Mycobacterium* strains. (A) *Mycobacterium tuberculosis* H37Rv, (B) *M. tuberculosis* H37Rv Δ*papA1*, (C) *M. tuberculosis* CDC1551, (D) *Mycobacterium bovis* Ravenel, (E) "*Mycobacterium canettii*" 140010060, (F) "*M. canettii*" 140010061, (G) *Mycobacterium kansasii* Hauduroy, (H) *M. kansasii* Hauduroy Δ*MKAN27435*. Lipid fractions were run as previously described by Dobson *et al*., 1985[1] using the following solvent systems:

(**a**) System C. Molybdophosphoric acid detection: *all lipids*.
Direction 1: Chloroform/methanol (96:4, v/v, once);
Direction 2: Toluene/acetone (80:20, v/v, once)

(**b**) System D. Molybdophosphoric acid detection: *all lipids*.
Direction 1: Chloroform/methanol/water (100:14:0.8, v/v/v, once);
Direction 2: Chloroform/acetone/methanol/water (50:60:2.5:3, v/v/v/v, once).

Abbreviations: FA, free fatty acids; MA, free mycolic acids; PGL(1), single sugar phenolic glycolipid; PGL(3), three sugar phenolic glycolipid; PGL(4), four sugar phenolic glycolipid; SGL, sulfoglycolipid; TDM, trehalose dimycolate.

Supplementary Figure S3.

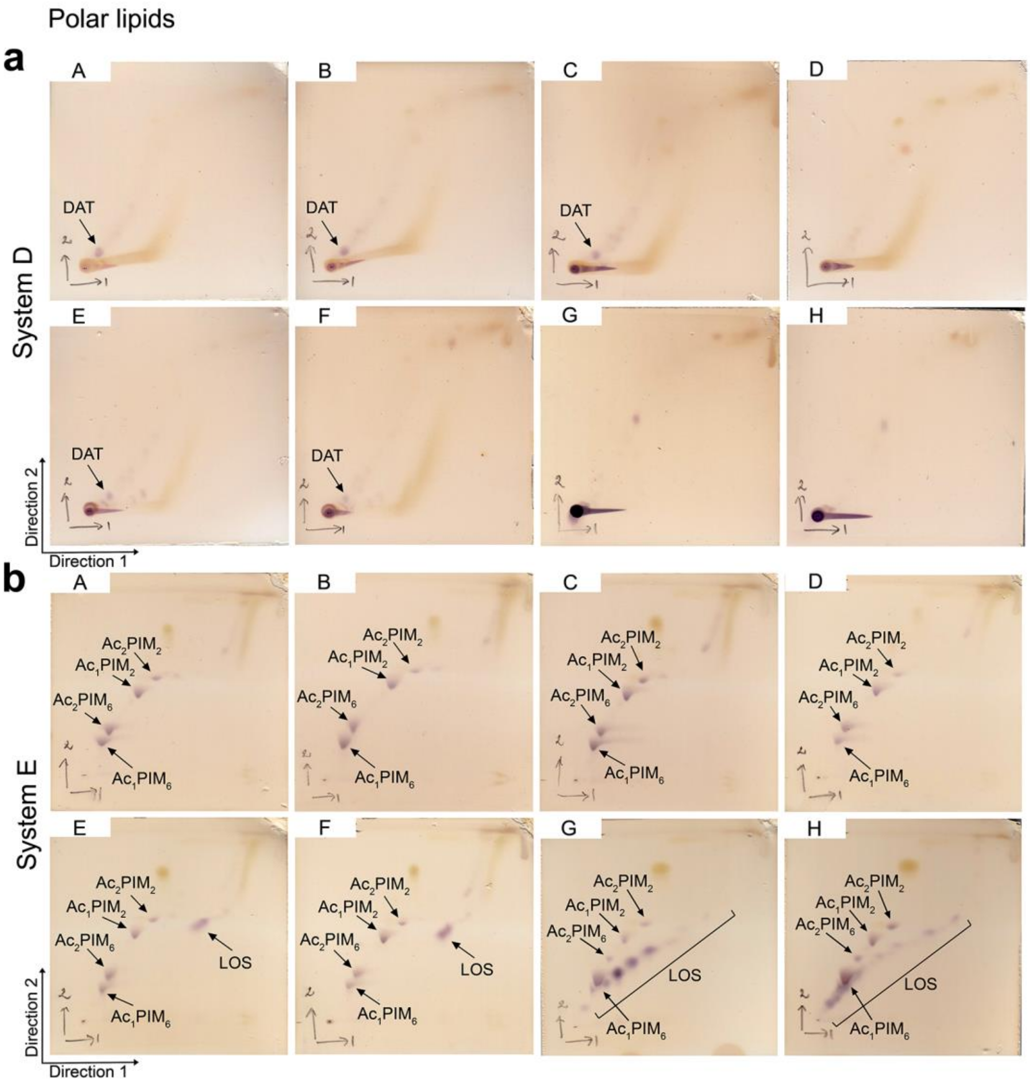

Supplementary Figure S3. Thin layer chromatography (TLC) patterns of polar lipid fractions from various *Mycobacterium* strains. (A) *Mycobacterium tuberculosis* H37Rv, (B) *M. tuberculosis* H37Rv Δ*papA1*, (C) *M. tuberculosis* CDC1551, (D) *Mycobacterium bovis* Ravenel, (E) "*Mycobacterium canettii*" 140010060, (F) "*M. canettii*" 140010061, (G) *Mycobacterium kansasii* Hauduroy, (H) *M. kansasii* Hauduroy Δ*MKAN27435*. Lipid fractions were run as previously described by Dobson *et al*., 1985[1] using the following solvent systems:

(**a**) System D. α-Naphthol-sulfuric acid detection: *glycolipids*.

Direction 1: Chloroform/methanol/water (100:14:0.8, v/v/v, once);

Direction 2: Chloroform/acetone/methanol/water (50:60:2.5:3, v/v/v/v, once).

(**b**) System E. α-Naphthol-sulfuric acid detection: *glycolipids*.

Direction 1: Chloroform/methanol/water (60:30:6, v/v/v, once);

Direction 2: Chloroform/acetic acid/methanol/water (40:25:3:6, v/v/v/v, once).

Abbreviations: DAT, diacyl trehaloses; LOS, lipooligosaccharides; $Ac_{1,2}PIM_{2,6}$, mono- and diacyl phosphatidylinositol di- and hexamannosides.

Supplementary Figure S4.

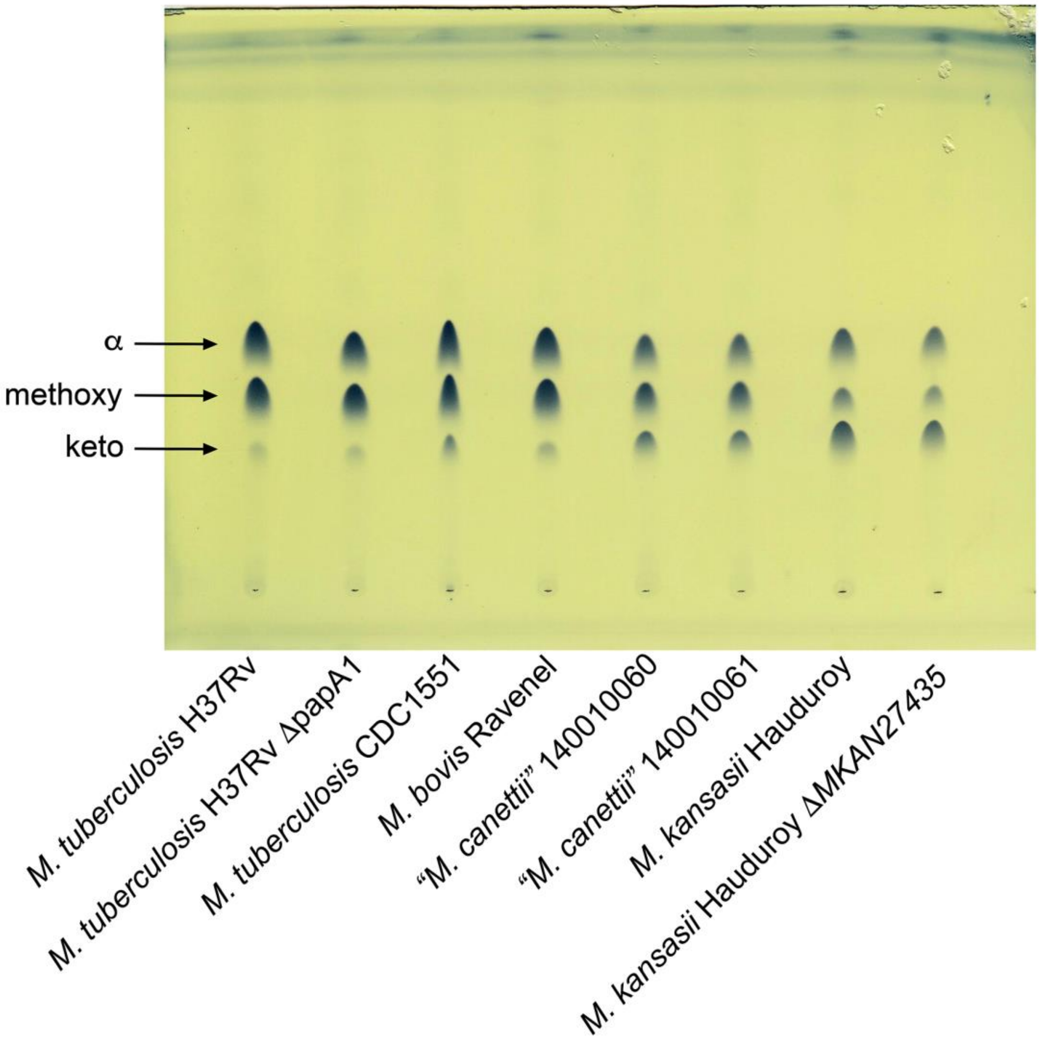

Supplementary Figure S4. Thin layer chromatography (TLC) of methyl esters of α-, methoxy- and ketomycolic acids from *Mycobacterium* strains. The chromatogram was developed thrice in petroleum ether/diethyl ether (90:10 v/v) and separated components revealed with molybdophosphoric acid.

REFERENCES

1. Dobson, G. *et al*. Systematic analysis of complex mycobacterial lipids. in *Chemical Methods in Bacterial Systematics.* (eds. Goodfellow M. & Minnikin D. E.) 237–265 (Academic Press, 1985).
